# How Rapidly Does the FAPI PET Signal Reverse Following Therapy? Assessing the FAPI PET Signal in Hypertensive Cardiac Injury and Fibrosis in Mice

**DOI:** 10.2967/jnumed.124.268860

**Published:** 2025-11

**Authors:** Atefeh Hosseini, Elias Haj-Yehia, Sebastian Korste, Yalcin Kuzay, Marija Trajkovic-Arsic, Stephan Settelmeier, Miriam Cantore, Katja B. Ferenz, Jens T. Siveke, Ken Herrmann, Tienush Rassaf, Ulrike Hendgen-Cotta, Wolfgang A. Weber, Zohreh Varasteh

**Affiliations:** 1 Department of Nuclear Medicine, Medical Faculty, University Hospital Essen, University of Duisburg-Essen, Essen, Germany;; 2 Department of Cardiology and Vascular Medicine, West German Heart and Vascular Center, Medical Faculty, University Hospital Essen, University of Duisburg-Essen, Essen, Germany;; 3 Bridge Institute of Experimental Tumor Therapy, West German Cancer Center, University Hospital Essen, University of Duisburg-Essen, Essen, Germany;; 4 German Cancer Consortium, partner site Essen, a partnership between German Cancer Research Center and University Hospital Essen, Essen, Germany;; 5 Institute of Physiology, University Hospital Essen, University of Duisburg-Essen, Essen, Germany; and; 6 Department of Nuclear Medicine, Klinikum rechts der Isar der TUM, Munich, Germany

**Keywords:** FAPI PET, signal reversibility, rapid therapy response monitoring

## Abstract

Reactive fibrosis is a complex response to chronic myocardial insults, contributing to heart failure progression. Fibroblast activation protein inhibitor (FAPI) PET shows promise in distinguishing active from established fibrosis. Although antifibrotic therapies may improve left ventricular (LV) function in preclinical studies, their clinical application is limited by the lack of noninvasive imaging methods to assess fibrosis regression. This study investigates the potential of FAPI PET to track the therapeutic transition of activated fibroblast activation protein (FAP)–positive fibroblasts toward a FAP-negative phenotype. **Methods:** Mice were implanted with minipumps, infused with angiotensin-II/phenylephrine (Ang-II/PE) for 6 wk and scanned with ^68^Ga-FAPI-46 PET/CT longitudinally. Control mice received saline. ^68^Ga-FAPI-46 biodistribution studies were conducted at preselected time points, and FAPI uptake in the major organs was measured ex vivo. To assess the potential reversibility of the FAPI PET signal in the myocardium and liver, Ang-II/PE infusion was discontinued in a group of animals at 1 and 2 wk, respectively. LV structural and functional changes were assessed via echocardiography, tissue fibrosis via histology, and FAP expression via immunohistochemistry. **Results:** Significant ^68^Ga-FAPI-46 uptake in the myocardium of treated mice peaked at 1 wk. An increase of ^68^Ga-FAPI-46 uptake was also observed in the liver, peaking at 2 wk, and decreased significantly at 4 wk. The PET signal declined to an indiscernible level in the heart and liver early after Ang-II/PE withdrawal. Three weeks after the removal of the minipumps, the hearts of mice previously exposed to Ang-II/PE for 1 wk exhibited a significant reduction in fibrosis compared with mice that were sacrificed immediately after 1 wk of Ang-II/PE infusion, without the 3-wk recovery period. Coinjection with excess unlabeled FAPI-46 reduced uptake in the heart, liver, and kidneys. Despite an increase in LV wall thickness at 1 wk, the ejection fraction remained stable initially but dropped significantly by 4 wk. **Conclusion:** The rapid decline in PET signal after Ang-II/PE withdrawal shows that FAPI PET effectively visualizes dynamic changes in FAP expression, making it a valuable tool for quickly assessing treatment responses targeting activated fibroblasts. The cardiac FAPI signal precedes functional myocardial changes, indicating that FAPI PET could detect early fibrosis in cardiac remodeling leading to heart failure. FAPI PET may also visualize cardiac cirrhosis, a serious complication of cardiac disorders.

## Visual Abstract

**Figure:**
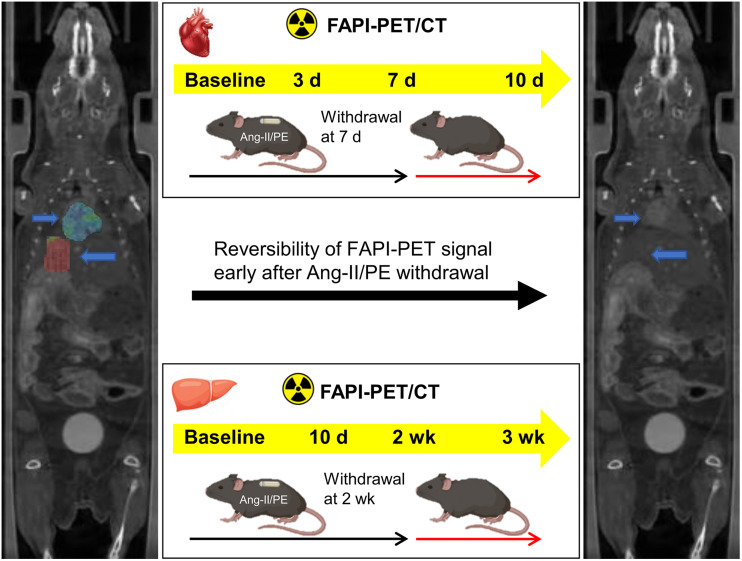

Cardiac remodeling involves changes at the molecular, cellular, and interstitial levels of the myocardium, resulting in structural and functional alterations in response to various myocardial conditions such as injury or stress. This process is recognized as a significant determinant in the progression of heart failure (HF) (1). Central to cardiac remodeling is myocardial fibrosis (2), characterized by the excessive accumulation of connective tissue components, such as collagen and other fibrillar extracellular matrix proteins in the cardiac interstitium, which distorts the normal architecture and function of the heart (3,4).

Fibrosis, once considered a persistently progressing and irreversible process, is now recognized as a dynamic process with potential for reversibility under certain circumstances (5–7). Activated fibroblasts are the primary cellular agents in myocardial fibrosis, functioning as secretory, extracellular matrix–producing, and contractile cells (8).

Although several antifibrotic therapies show promise for improving cardiac function in preclinical settings (9–12), the path toward clinical translation is hampered by the lack of established noninvasive imaging techniques to assess fibrosis regression in response to therapy (13).

The fibroblast activation protein (FAP) is a biomarker that is selectively overexpressed on activated fibroblasts (14–17). Emerging FAP-targeted PET imaging methods using radiolabeled FAP inhibitors (FAPI) has shown high sensitivity and specificity in detecting active fibroblasts (18–21). However, it remains unknown whether FAPI PET can demonstrate the reversibility of fibroblast activation and if the fibroblasts revert to a quiescent state in response to therapeutic interventions.

This study aims to explore the potential of FAPI PET in evaluating cardiac remodeling, with a focus on its ability to assess dynamic changes in FAP expression in a mouse model of hypertensive heart disease and the reversion of FAP-positive fibroblasts to a FAP-negative state after the discontinuation of hypertension-inducing agents. FAPI PET signal reduction may indicate the speed at which active fibroblasts transition to a quiescent state in response to therapy.

## MATERIALS AND METHODS

### Angiotensin-II/Phenylephrine (Ang-II/PE) Model of Hypertensive Cardiac Injury and Fibrosis in Mice

In total, 54 male C57BL/6J mice, weighing 26.3 ± 1.8 g, were purchased from Janvier Labs.

Osmotic minipumps (ALZET-2006; ALZET Osmotic Pumps) with a 200-μL capacity were filled with a solution of angiotensin-II (Ang-II, HY-13948; MedChem Express) infused at a rate of 1.5 mg/kg/d and (*R*)-(−)-phenylephrine hydrochloride (PE, P6126-10G; Sigma-Aldrich) infused at a rate of 50 mg/kg/d. The pumps were implanted subcutaneously beneath the dorsal skin of the mice. Control mice received saline. The experiments were approved by the local animal care committee and were in accordance with the German Animal Welfare Act (Landesregierung von Nordrhein-Westfalen).

### Tracer Production

The radiolabeling of FAPI-46 was adapted from an established procedure (22). Details are provided in the supplemental material (available at http://jnm.snmjournals.org).

### ^68^Ga-FAPI-46 PET/CT Imaging

Mice were injected intravenously with ^68^Ga-FAPI-46 (∼100 µL, ∼10 MBq, 540 ± 30 pmol). PET and CT images were acquired in β-CUBE and X-CUBE (Molecubes), respectively. Imaging was performed at 45 ± 5 min after injection. To quantify tracer uptake, spheric volumes of interest with a radius of 2 mm were placed in the heart, liver, and kidneys, and either the average SUV_mean_ or the SUV_mean_ without average of FAPI was calculated.

### In Vivo Binding Specificity Test

To evaluate the specificity of ^68^Ga-FAPI-46 accumulation and confirm that its uptake in the heart, liver, and kidneys was due to saturable binding to FAP, a group of mice (*n* = 3) was coinjected with a blocking dose (30 nmol) of nonlabeled FAPI-46 after 2 wk of Ang-II/PE infusion. The same animals had been scanned 1 d earlier with ^68^Ga-FAPI-46 (480–500 pmol) PET/CT without the blocking compound injection.

### Echocardiography

Mouse echocardiography was performed using a VisualSonics Vevo ultrasound system. 2-Dimensional B- and M-mode cine loop images were acquired in the parasternal long and parasternal short-axis views, as well as the apical 4-chamber view. M-mode images in the short-axis view were recorded at the midpapillary left ventricle level. Postanalysis was performed using VevoLab 3.2 software (FUJIFILM VisualSonics).

### Biodistribution

A biodistribution study of ^68^Ga-FAPI-46 was performed at baseline, 1, 2, and 6 wk after Ang-II/PE infusion. Mice were euthanized 45 ± 5 min after intravenous injection, major organs were harvested, and radioactivity was measured using a γ-counter (2480 WIZARD2; PerkinElmer). Data were normalized to tissue weight and expressed as percentage of injected radioactivity per gram.

### Histology and Immunohistochemistry

Histology and immunohistochemistry were conducted on formalin-fixed, paraffin-embedded tissue samples following standard laboratory protocols. Details are provided in the supplemental materials.

### Light Sheet Microscopy

For whole-mount staining of cardiac FAP expression, we followed a modified version of a previously published protocol (23). Details can be found in the supplemental materials.

### Statistical Analysis

Data are presented as mean ± SD. The Wilcoxon rank-sum test was used for comparing 2 independent variables, whereas the Wilcoxon signed-rank test was applied for paired measurements. A *P* value of 0.05 or less was considered significant. All statistical analyses were performed using JMP Pro 18 (JMP Statistical Discovery).

## RESULTS

FAPI-46 was labeled with ^68^Ga, achieving an overall radiochemical purity of 99.3% ± 0.2% and a specific activity of approximately 0.03 MBq/pmol.

### Assessing Dynamics of Whole-Body Fibroblast Activation in Mice in Response to Ang-II/PE Through Longitudinal ^68^Ga-FAPI-46 PET/CT Imaging

**FIGURE 1.**
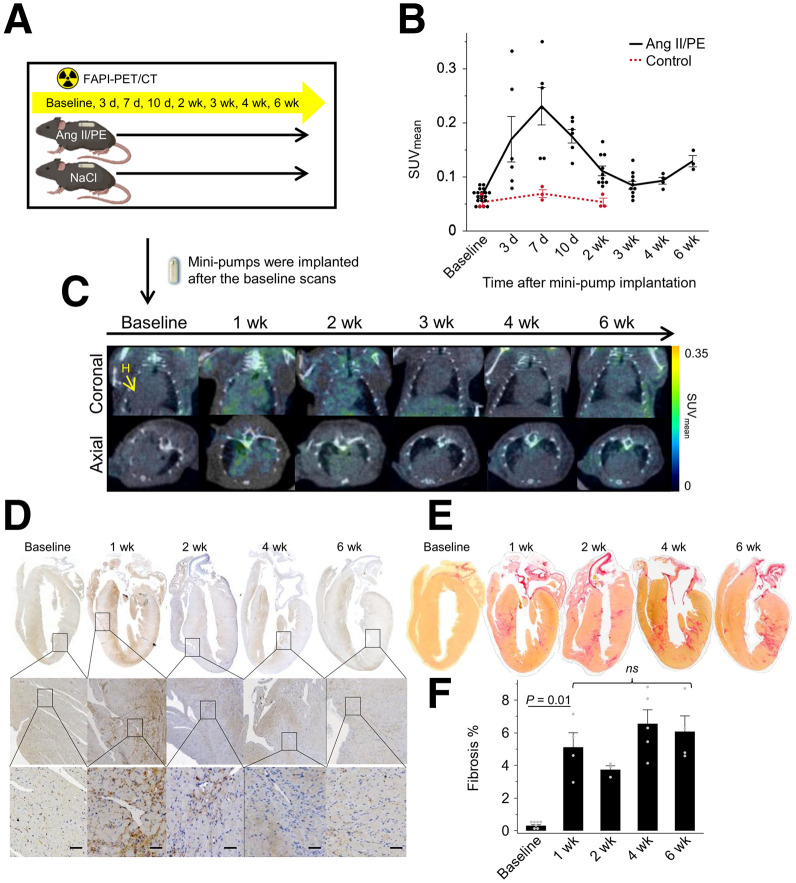
FAPI uptake, FAP expression, and fibrosis in myocardium of Ang-II/PE–infused mice in longitudinal study. (A) Experimental schematic for in vivo FAPI PET/CT imaging of cardiac fibroblast activation in myocardial tissue. C57BL/6J mice were continuously infused with either Ang-II/PE to induce cardiac injury and fibrosis or saline (control) for 6 wk via osmotic minipump and were scanned with FAPI PET. (B) Corresponding time–activity curves of cardiac tissue for Ang-II/PE–infused and control groups. To quantify tracer uptake, spheric volumes of interest with radius of 2 mm were positioned to cover whole heart. (C) Static PET/CT coronal and axial slices from same Ang-II/PE–infused mouse at different time points, with focus on heart signal. (D) Immunostaining for FAP (scale bar = 50 μm). Zoomed-in images were intentionally chosen from regions with extensive fibrosis (intense Picrosirius-Red staining). (E) Picrosirius-Red staining of whole hearts. (F) Quantification of cardiac fibrotic area (ratios of Picrosirius-Red staining area to total left ventricle area presented as fibrosis %). ns = not significant.

One week after minipump implantation, Ang-II/PE–infused mice showed significantly elevated myocardial ^68^Ga-FAPI-46 uptake compared with control mice (SUV_mean_: 0.23 ± 0.08 vs. 0.06 ± 0.01, *P* = 0.001) (Fig. 1). Ex vivo analysis confirmed higher cardiac uptake compared with baseline (percentage of injected radioactivity per gram: 1.9 ± 0.4 vs. 0.11 ± 0.02, *P* = 0.04) (Table 1). This uptake then gradually declined to baseline levels. Immunohistochemistry corroborated a transient expression of FAP in the myocardium, irrespective of ongoing insult (Fig. 1D). A significant increase in cardiac fibrosis occurred within the first week of Ang-II/PE infusion, after which the progression was gradual and not statistically significant (Figs. 1E and 1F). Supplemental Figure 1 displays the 3-dimensional regional distribution of FAP in the heart of a mouse infused with Ang-II/PE for 1 wk as well as a baseline heart. FAP expression was primarily observed within or bordering less autofluorescent tissue regions, likely indicating damaged areas, as these were absent in the baseline sample.

**TABLE 1.** Biodistribution Study Results*

Tissue (%IA/g)	Baseline	1 wk	2 wk	6 wk
Blood	0.57 ± 0.05	0.6 ± 0.2	0.6 ± 0.1	1.09 ± 0.36
Heart	0.11 ± 0.02	1.9 ± 0.4^acd^	0.30 ± 0.05	0.6 ± 0.3
Liver	1.1 ± 0.3	3.3 ± 0.4	10.6 ± 2.1^be^	2.5 ± 0.4
Kidney	1.8 ± 0.2	4.9 ± 0.7^a^	4.3 ± 1.5	8.1 ± 1.8
Muscle	0.16 ± 0.03	0.36 ± 0.04	0.2 ± 0.1	0.16 ± 0.07

*Superscript lowercase letters denote statistically significant differences (*P* ≤ 0.05) where value indicated by letter is higher than comparison group. ^a^Baseline vs. 1 wk, ^b^baseline vs. 2 wk, ^c^1 wk vs. 2 wk, ^d^1 wk vs. 6 wk, ^e^2 wk vs. 6 wk.

%IA/g = percentage of injected radioactivity per gram.

Data are presented as mean values ± SD (*n* = 3).

**FIGURE 2.**
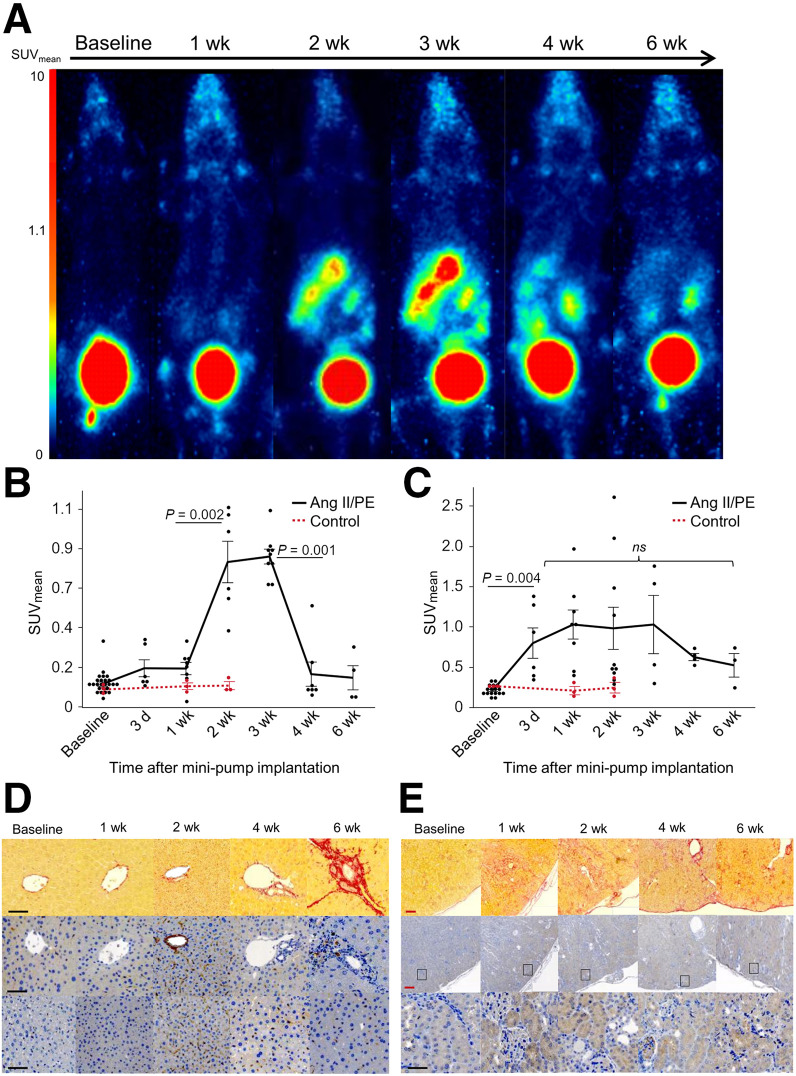
FAPI uptake, FAP expression, and fibrosis in liver and kidneys of Ang-II/PE–infused mice in longitudinal study. (A) Maximum-intensity projection PET images from same mouse (Ang-II/PE–infused) at different time points. Corresponding time–activity curves of liver (B) and kidneys (C) for Ang-II/PE–infused and control groups. Control animals infused with saline. (D) Top, Picrosirius-Red staining; center, perivascular FAP; bottom, interstitial FAP expression in liver tissue. (E) Top, Picrosirius-Red staining; center and bottom, FAP expression in kidneys. Red scale bar = 200 µm; black scale bar = 50 µm. ns = not significant.

Elevated ^68^Ga-FAPI-46 uptake was also observed in the liver and kidneys (Fig. 2A). Compared with that in control mice, a significant hepatic signal originated from the left lateral lobe of the liver (2 wk SUV_mean_, 0.12 ± 0.03 vs. 0.81 ± 0.26; *P* = 0.01) and spread throughout the liver over the following weeks (Supplemental Fig. 2). The liver uptake remained elevated until 3 wk (SUV_mean_, 0.84 ± 0.12) and significantly decreased by 4 wk (SUV_mean_, 0.18 ± 0.16; *P* = 0.001; Fig. 2B).

Early after infusion, renal uptake was significantly increased in the Ang-II/PE–treated group compared with controls (1 wk SUV_mean_, 1.03 ± 0.05 vs. 0.21 ± 0.09; *P* = 0.01; Fig. 2C).

Immunohistochemistry analysis revealed increased FAP expression in the liver, both perivascular and interstitial, at 2 wk after Ang-II/PE infusion, leading primarily to perivascular fibrosis (Fig. 2D). Relatively intense fibrosis was observed in the renal parenchyma of mice treated with Ang-II/PE compared with baseline kidneys (Fig. 2E). FAP expression was observed in the glomeruli and tubular cells within the renal cortex of Ang-II/PE–treated kidneys at all time points except baseline (Fig. 2E). Histologic findings indicated that Ang-II/PE primarily induced abnormalities in glomerular tuft, tubules, and Bowman capsule space (Supplemental Fig. 3).

**FIGURE 3.**
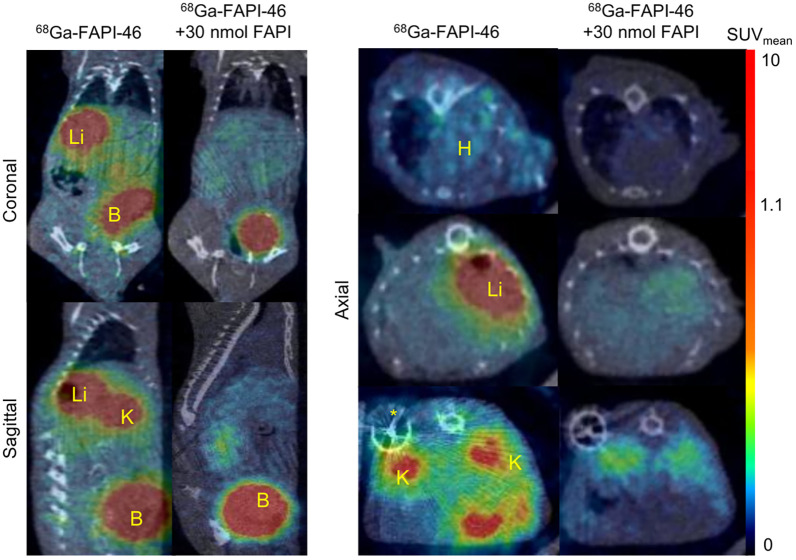
Binding specificity test. Representative PET/CT coronal, sagittal, and axial views of same mouse without and with coinjection with 30 nmol unlabeled FAPI-46. PET/CT images from nonblocked mouse show increased ^68^Ga-FAPI-46 uptake in heart, liver, and kidneys of mouse infused with Ang-II/PE for 2 wk, whereas uptake is negligible after injection of unlabeled FAPI-46 (blocked). Same animals had been scanned 1 d earlier with ^68^Ga-FAPI-46 PET/CT (480–500 pmol, 8–10 MBq) without injection of blocking compound. Yellow asterisk marks position of minipump, which creates artifacts in the CT images. B = bladder; H = heart; Li = liver; K = kidney.

After 2 wk of Ang-II/PE infusion, coinjection of a nearly 60-fold excess of nonlabeled FAPI-46 to saturate FAP resulted in a significant reduction in uptake in the heart (SUV_mean_, from 0.11 ± 0.03 to 0.05 ± 0.02; *P* = 0.01), liver (SUV_mean_, from 0.81 ± 0.26 to 0.26 ± 0.12; *P* = 0.01), and kidneys (SUV_mean_, from 0.9 ± 0.8 to 0.3 ± 0.1; *P* = 0.03) (Fig. 3).

The initial longitudinal PET study aimed to determine the onset of FAPI signal after Ang-II/PE infusion. On the basis of the dynamics of the PET signal observed in the longitudinal study, weeks 1 and 2 were selected to discontinue Ang-II/PE infusion in groups of animals to evaluate the potential reversibility of the FAPI PET signal in the myocardium and liver, respectively.

### Cardiac Hypertrophy and Dysfunction in Mice Treated with Ang-II/PE

**FIGURE 4.**
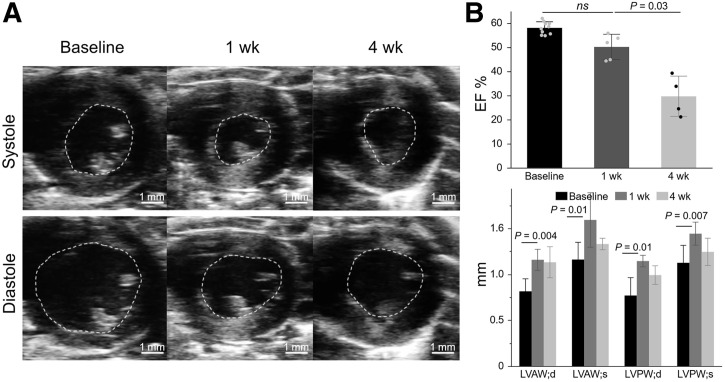
Echocardiography. (A) Representative 2-dimensional short-axis B-mode systolic and diastolic views taken at baseline, as well as 1 wk and 4 wk after Ang-II/PE infusion. (B) Echocardiogram analysis results of Ang-II/PE–infused mice. Scale bar = 1 mm for all images. EF = ejection fraction; LVAW;d = left ventricular anterior wall thickness end diastole; LVAW;s = left ventricular anterior wall thickness end systole; LVPW;d = left ventricular posterior wall thickness end diastole; LVPW;s = left ventricular posterior wall thickness end systole; ns = not significant.

Functional and structural echocardiographic data over time in mice infused with Ang-II/PE are shown in Figure 4. Despite a substantial increase in the thickness of the left ventricle anterior and posterior walls at 1 wk (when the FAPI PET signal in the myocardium was at its peak), the ejection fraction remained stable (56.0 ± 1.5% at baseline vs. 51.5% ± 4.3% at 1 wk, *P* = 0.1). The ejection fraction then dropped significantly as HF initiated and progressed at 4 wk (29.8% ± 7.2%, *P* = 0.03).

### Rapid Reversibility of the FAPI PET Signal After Ang-II/PE Withdrawal

In a subset of animals, minipumps were removed at 7 d to evaluate the reversibility of myocardial fibroblast activation and at 2 wk to assess reversibility in the liver.

**FIGURE 5.**
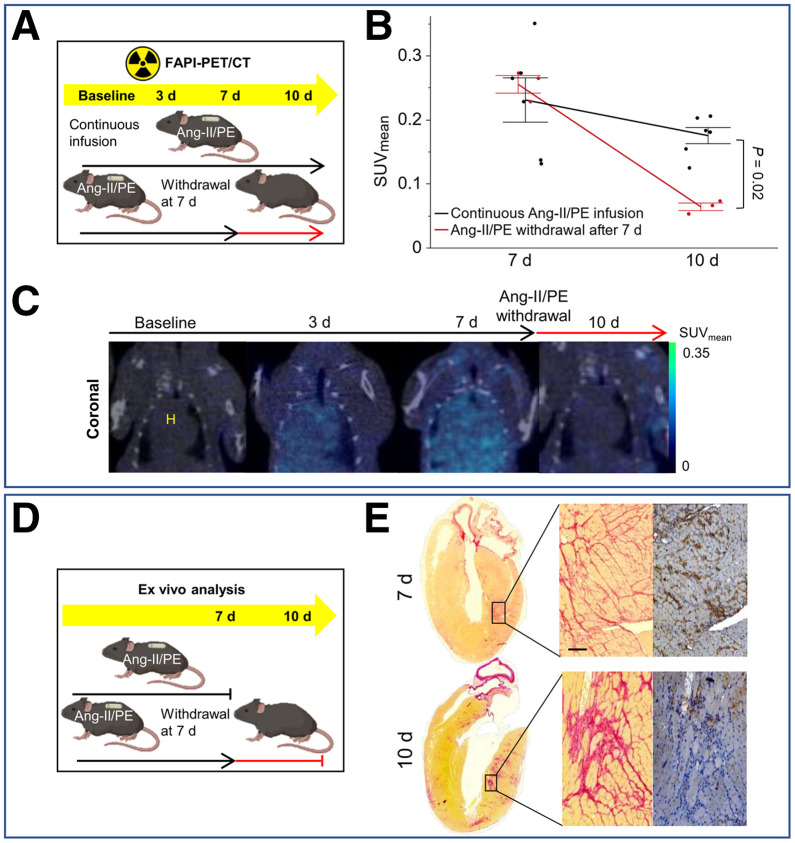
Reversibility of FAPI PET signal and FAP expression in myocardial tissue early after Ang-II/PE withdrawal. (A) Experimental design for in vivo FAPI PET imaging to monitor myofibroblast deactivation after cessation of hypertension-inducing agents. Group of C57BL/6J mice (*n* = 6) received continuous Ang-II/PE treatment for up to 10 d. Another group (*n* = 3) was given Ang-II/PE for 7 d using osmotic minipump, which was then removed after this period. (B) Time–activity curves for cardiac tissue are provided for mice continuously infused with Ang-II/PE for 10 d and for those with Ang-II/PE infusion discontinued after 7 d. (C) Static PET/CT coronal slices from same mouse at different time points, focusing on heart signals. Mouse was infused with Ang-II/PE for 7 d, followed by a 3-d cessation of infusion. To quantify tracer uptake, spheric volumes of interest with radius of 2 mm were used to cover whole heart. (D) Experimental design for ex vivo FAP expression to monitor myofibroblast deactivation after cessation of hypertension-inducing agents. Group of mice (*n* = 3) sacrificed immediately after 7 d of Ang-II/PE treatment. Another group (*n* = 3) was sacrificed after 1 wk of Ang-II/PE infusion plus 3-d withdrawal period (10 d). (E) Histology and immunohistochemistry. Scale bar = 50 µm.

**FIGURE 6.**
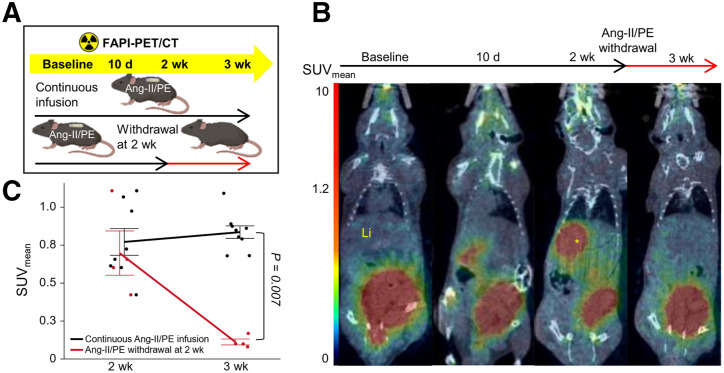
Reversibility of the FAPI PET signal in liver tissue early after Ang-II/PE withdrawal. (A) Experimental design for in vivo FAPI PET imaging to monitor deactivation of active hepatic fibroblasts after discontinuation of hypertension-inducing agents. Group of C57BL/6J mice (*n* = 9) received continuous Ang-II/PE treatment for up to 3 wk. Another group (*n* = 4) was given Ang-II/PE for 2 wk using osmotic minipump, which was then removed after this period. (B) Static PET/CT coronal slices from same mouse at different time points, focusing on liver signal. Mouse was infused with Ang-II/PE for 2 wk, followed by 1-wk cessation of infusion. To quantify tracer uptake, spheric volumes of interest with radius of 2 mm were placed in left lateral lobe of liver, where signal first originated. Yellow asterisk indicates position of spheric volumes of interest. (C) Time–activity curves for liver tissue are provided for mice continuously infused with Ang-II/PE for 3 wk and for those with Ang-II/PE infusion discontinued after 2 wk.

In the heart (Fig. 5), the SUV_mean_ was significantly lower in animals with a 3-d withdrawal period compared with those with continuous 10-d infusion (0.06 ± 0.01 vs. 0.18 ± 0.03, *P* = 0.02) (Fig. 5B). After the withdrawal of hypertension-inducing agents, the FAPI PET signal in the myocardium returned to its baseline level within 3 days (Fig. 5C). Ex vivo analysis of cardiac tissue (Fig. 5E) indicated that, despite substantial myocardial fibrosis, FAP expression was relatively lower in animals with 3 d of rest. Similarly, in the liver (Fig. 6), SUV_mean_ was significantly lower in animals with 1 wk of rest compared with those with continuous 3-wk infusion (0.11 ± 0.03 vs. 0.81 ± 0.26, *P* = 0.007).

### Reversibility of Fibrosis After Ang-II/PE Withdrawal

**FIGURE 7.**
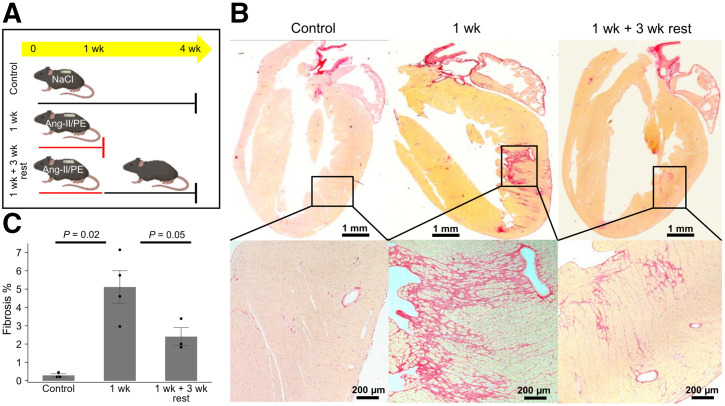
Reversibility of fibrosis after Ang-II/PE withdrawal. (A) Experimental design for ex vivo staining of collagen deposits to observe reversal of fibrosis after discontinuation of hypertension-inducing agents. Control group (*n* = 3) received saline for up to 4 wk. One group (*n* = 4) received Ang-II/PE for 1 wk and was sacrificed immediately afterward. Another group (*n* = 3) received Ang-II/PE infusion for 1 wk, which was then discontinued. These animals were sacrificed after a 3-wk rest period. (B) Top, Picrosirius-Red staining of whole heart; coronal sections in mice treated with saline (left), Ang-II/PE infusion for 1 wk (center), or 1 wk of Ang-II/PE and 3-wk rest period (right) to evaluate fibrosis (red). Bottom, magnification of left ventricular fibrosis. (C) Quantification of cardiac fibrosis.

Significantly increased cardiac fibrosis was observed, primarily in the left ventricular wall, in mice 1 wk after Ang-II/PE infusion compared with controls (Fig. 7). Three weeks after discontinuing Ang-II/PE, a significant reduction in collagen deposits was noted in the previously injured mice that had been infused with Ang-II/PE for 1 wk.

## DISCUSSION

We reproduced a well-established model of Ang-II/PE–induced cardiac remodeling in male C57BL/6J mice (24) to evaluate the feasibility of monitoring cardiac fibroblast activation in this hypertensive mouse model using ^68^Ga-FAPI-46 and to assess the reversibility of the FAPI PET signal and fibrosis in response to therapy (withdrawal of Ang-II/PE). Peak cardiac FAP expression and ^68^Ga-FAPI-46 uptake occurred 1 wk after minipump implantation, followed by a decline with the onset and progression of HF. Interestingly, a distinct FAPI signal was also detected in the liver of mice 2 wk after continuous Ang-II/PE infusion. The liver uptake remained elevated until 3 wk after infusion before subsequently declining. Both cardiac fibroblast activation and cardiac fibrosis were reversible after Ang-II/PE withdrawal. Very early after Ang-II/PE withdrawal, the cardiac FAPI PET signal dropped to the baseline level. Three weeks after removing the minipumps, the injured hearts of mice that had been exposed to Ang-II/PE for 1 wk showed a significant reduction in interstitial fibrosis compared with the mice that were sacrificed immediately after 1 wk of Ang-II/PE infusion without a 3-wk rest period. Similar to the myocardium, the withdrawal of Ang-II/PE led to a significant decline in the hepatic FAPI PET signal. A blockable FAPI PET signal was also detected in the kidneys of mice infused with Ang-II/PE. All 3 organs have been found to lack FAP activity in healthy mice (Figs. 1 and 2) (25).

Hypertensive heart disease, currently the second leading cause of HF, advances through various mechanisms (26). Sustained elevated blood pressure results in endothelial dysfunction, reduced coronary blood flow, and decreased capillary density. These adverse factors restrict oxygen transport in the heart and accelerate cardiac remodeling. Additionally, high blood pressure can lead to arterial stiffness, placing excessive strain on the heart and further exacerbating the problem by impairing contraction and diastolic function, resulting in HF. Early identification and treatment of cardiac remodeling before manifestation of HF is crucial for better prognosis. The transition from compensated remodeling to decompensated HF is frequently irreversible, underscoring the importance of early intervention to halt disease progression and maintain cardiac function. The clinical diagnosis of cardiac remodeling is primarily relying on detecting morphologic changes, such as alterations in size, mass, geometry, and function after injury using echocardiography. Molecular changes, however, may occur earlier. Unlike echocardiography, which primarily captures macroscopic structural and functional changes, molecular imaging of FAP expression may visualize fibroblast activation before overt morphologic or functional deterioration occurs. Like in the rat model of isoproterenol-induced HF (20), we observed that the cardiac FAPI PET signal emerged before echocardiography detected reduced cardiac function, suggesting that FAPI PET could serve as a valuable tool for visualizing the initial phases of fibrosis in HF.

Among the infusion substances used in this study, PE is a selective α1-adrenergic receptor agonist that increases blood pressure by raising systemic vascular resistance without directly impacting myocardial contractility (27). The α1-receptors primarily mediate contraction of smooth muscle cells, and activation of these receptors in blood vessels results in vasoconstriction (28). On the other hand, the effect of Ang-II, a multifunctional peptide hormone and principal effector molecule of the renin–angiotensin system, on its target organs occurs through 2 types of receptors: type 1 and type 2 receptors (AT1R and AT2R). AT1R primarily mediates the major effects of Ang-II. In the cardiovascular system, Ang-II causes a significant increase in transforming growth factor β1 messenger RNA in cardiomyocytes and cardiac fibroblasts, which the latter transdifferentiate to myofibroblast phenotype resulting in myocardial fibrosis (29). The direct mechanisms responsible for cardiac fibrosis and the effector cells involved in response to Ang-II infusion have not yet been fully characterized.

The pathophysiology of cardiac cirrhosis is characterized by elevated hepatic venous pressure, reduced hepatic perfusion, and diminished arterial oxygen saturation. Moreover, the liver harbors numerous Ang-II binding sites, and activation of the renin–angiotensin system, by stimulating AT1R on hepatic stellate cells, is known to contribute to fibrogenesis (30,31). Therefore, fibroblast activation in the liver in our model may partially result from a direct Ang-II/AT1R–mediated mechanism. However, Wang et al. investigated FAPI PET imaging in a pressure overload–induced HF model over an 8-wk period without any pharmacologic intervention (32). In their study, Sprague–Dawley rats subjected to abdominal aortic constriction displayed a transient hepatic FAPI signal that peaked at 4 wk and declined thereafter, despite the persistence of insult. This finding suggests that the hepatic FAPI signal may not only stem from direct pharmacologic stimulation but could also reflect interorgan fibrotic link—likely secondary to hepatic congestion due to impaired cardiac function.

The rapid decrease in the FAPI PET signal after Ang-II/PE withdrawal demonstrates that ^68^Ga-FAPI may be suitable for rapidly assessing treatment responses aimed at eliminating activated fibroblasts. However, extrapolating these research data to humans requires caution, as fibrosis development in humans is a much slower process compared with mice, often taking decades to develop (13). The process of silencing active fibroblasts may also be slower in humans, necessitating long-term treatment to reduce fibrosis progression and the associated FAPI PET signal. Additionally, the animals used in this study are young, whereas patients with cardiac fibrosis are at a more advanced age. Nonetheless, a key takeaway from these findings is that the FAPI PET signal highlights the initiation and early stages of cardiac remodeling, a phase when treatments may be more effective.

Our study, while showing FAP expression dynamics in multiple mouse tissues over a 6-wk period, has a limitation: we did not investigate whether activated fibroblasts revert to a quiescent state or cease FAP expression as they mature into more advanced myofibroblasts during Ang-II/PE infusion.

## CONCLUSION

Rapid reversibility of the PET signal after Ang-II/PE withdrawal demonstrated that ^68^Ga-FAPI can visualize dynamic changes in FAP expression and may be suitable for rapid assessment of treatment response aimed at deactivation or elimination of myofibroblasts. The cardiac FAPI signal emerges before functional alterations in the myocardium, suggesting that FAPI PET could serve as a valuable tool for visualizing the initial phases of fibrosis in cardiac remodeling leading to HF. Cardiac cirrhosis is a well-known complication of cardiac disorders, and FAPI may also be able to visualize the complications of heart conditions.

## DISCLOSURE

This research was financially supported by German Research Foundation (Deutsche Forschungsgemeinschaft-DFG; VA 1183/2-1). Atefeh Hosseini is supported by the German Academic Exchange Service (Deutscher Akademischer Austauschdienst-DAAD). Jens Siveke receives honoraria as a consultant or for continuing medical education presentations from AstraZeneca, Bayer, Boehringer Ingelheim, Bristol Myers Squibb, Immunocore, MSD, Novartis, Roche/Genentech, and Servier. His institution receives research funding from Abalos Therapeutics, Boehringer Ingelheim, Bristol Myers Squibb, Celgene, Eisbach Bio, and Roche/Genentech; he holds ownership in FAPI Holding (<3%); all are outside the submitted work. No other potential conflict of interest relevant to this article was reported.

## Notes

KEY POINTS **QUESTION:** How quickly after therapy can FAPI PET depict the deactivation or elimination of active fibroblasts? **PERTINENT FINDINGS:** The rapid reversibility of the PET signal following Ang-II/PE withdrawal suggests that FAPI PET is effective for rapid assessment of treatment response aimed at deactivation or elimination of myofibroblasts. The cardiac FAPI signal emerges before functional changes in the myocardium, highlighting its potential as an early marker of cardiac remodeling that may lead to HF. **IMPLICATIONS FOR PATIENT CARE:** FAPI PET could potentially demonstrate therapy effectiveness without the need to wait for functional changes in the myocardium, allowing for quicker assessment of treatment outcomes.
